# Supplementary material for: Archaeal chromatin ‘slinkies’ are inherently dynamic complexes with deflected DNA wrapping pathways
Source: eLife. 2021 Mar 2;10:e65587. doi: 10.7554/eLife.65587 (PMC7990501; doi:10.7554/eLife.65587)
Supplement: Transparent reporting form [file elife-65587-transrepform.docx]

***eLife’s* transparent reporting form**

We encourage authors to provide detailed information *within their submission* to facilitate the interpretation and replication of experiments. Authors can upload supporting documentation to indicate the use of appropriate reporting guidelines for health-related research (see [EQUATOR Network](http://www.equator-network.org/%20)), life science research (see the [BioSharing Information Resource](https://biosharing.org/)), or the [ARRIVE guidelines](http://www.plosbiology.org/article/info:doi/10.1371/journal.pbio.1000412) for reporting work involving animal research. Where applicable, authors should refer to any relevant reporting standards documents in this form.

If you have any questions, please consult our Journal Policies and/or contact us: [editorial@elifesciences.org](mailto:editorial@elifesciences.org).

**Sample-size estimation**

- You should state whether an appropriate sample size was computed when the study was being designed
- You should state the statistical method of sample size computation and any required assumptions
- If no explicit power analysis was used, you should describe how you decided what sample (replicate) size (number) to use

Please outline where this information can be found within the submission (e.g., sections or figure legends), or explain why this information doesn’t apply to your submission:

Because the results of MD simulations are coupled to their initially assigned velocities, we run multiple replicates from the same initial coordinates, as described in the “Molecular Dynamics Simulations” section of the Methods section. Average quantities (such as RMSF or MM-GBSA-derived binding energies) are then compared across these independent trajectories (as we have previously done – Morrison et al., *eLife*, 2018). Statistical efficiency (and the effective number of non-correlated data points) for each timeseries was calculated using the timeseries module of pymbar, as noted in the “Simulation Analysis” methods section.

In SV-AUC experiments, the common practice is to conduct a pilot experiment, at what may be the less than ideal rotational speed or sample concentration, to determine the optimum experimental parameters for extracting the best signal-to-noise ratio per scan and number of radial scans to most smoothly determine the sedimentation profile. These pilot experiments identified the Mg-induced trend for Arc207 aggregation-less compaction, and the reported values and profiles are from the “production” experiment with optimized parameters.

Sample size efficiency in cryoEM experiments are typically determined through screening of grid conditions, followed by a long term collection. In the “CryoEM Grid Preparation and Collection” section, we outline that screening datasets were collected prior to our full-length collection for 3d particle analysis.

**Replicates**

- You should report how often each experiment was performed
- You should include a definition of biological versus technical replication
- The data obtained should be provided and sufficient information should be provided to indicate the number of independent biological and/or technical replicates
- If you encountered any outliers, you should describe how these were handled
- Criteria for exclusion/inclusion of data should be clearly stated
- High-throughput sequence data should be uploaded before submission, with a private link for reviewers provided (these are available from both GEO and ArrayExpress)

Please outline where this information can be found within the submission (e.g., sections or figure legends), or explain why this information doesn’t apply to your submission:

Three simulation replicates were run per system, as described in the “Simulation Analysis” section. CryoEM samples were screened and then 5,388 images were collected, as noted in the “CryoEM Grid Preparation and Collection” section.

**Statistical reporting**

- Statistical analysis methods should be described and justified
- Raw data should be presented in figures whenever informative to do so (typically when N per group is less than 10)
- For each experiment, you should identify the statistical tests used, exact values of N, definitions of center, methods of multiple test correction, and dispersion and precision measures (e.g., mean, median, SD, SEM, confidence intervals; and, for the major substantive results, a measure of effect size (e.g., Pearson's r, Cohen's d)
- Report exact p-values wherever possible alongside the summary statistics and 95% confidence intervals. These should be reported for all key questions and not only when the p-value is less than 0.05.

Please outline where this information can be found within the submission (e.g., sections or figure legends), or explain why this information doesn’t apply to your submission:

We outline our use of t-tests to compare simulation values in the “Simulation Analysis” section of our Methods, as well as in the legend of Table 1.

(For large datasets, or papers with a very large number of statistical tests, you may upload a single table file with tests, Ns, etc., with reference to sections in the manuscript.)

**Group allocation**

- Indicate how samples were allocated into experimental groups (in the case of clinical studies, please specify allocation to treatment method); if randomization was used, please also state if restricted randomization was applied
- Indicate if masking was used during group allocation, data collection and/or data analysis

Please outline where this information can be found within the submission (e.g., sections or figure legends), or explain why this information doesn’t apply to your submission:

CryoEM particle analysis identified 2 distinct classes of particles (we classify these as “Class I”, with ~150 bp of DNA and 5 histone dimers resolved, and “Class II”, with the appropriate volume to contain all 207 bp of DNA and 7 histone dimers), and the differentiation of these datasets is discussed in the methods, as well as Figure 6.

**Additional data files (“source data”)**

- We encourage you to upload relevant additional data files, such as numerical data that are represented as a graph in a figure, or as a summary table
- Where provided, these should be in the most useful format, and they can be uploaded as “Source data” files linked to a main figure or table
- Include model definition files including the full list of parameters used
- Include code used for data analysis (e.g., R, MatLab)
- Avoid stating that data files are “available upon request”

Please indicate the figures or tables for which source data files have been provided:

We have included source data for our figures in .csv file form, wherever possible. EM data has been deposited to the EMDB (EMD-23403, EMD-23404) will be released upon acceptance of the manuscript, and models of Class I and Class II have been included as supplementary PDB files. Raw trajectories of MD simulations correspond to over 2 TB of information, and cannot be easily shared in a repository, due to its size. These datasets will be stored locally and available upon request.
